# Presence of lymphocytic infiltrate cytotoxic T lymphocyte CD3^+^, CD8^+^, and immunoscore as prognostic marker in patients after radical cystectomy

**DOI:** 10.1371/journal.pone.0205746

**Published:** 2018-10-11

**Authors:** Alice Yu, Jose Joao Mansure, Shraddha Solanki, D. Robert Siemens, Madhuri Koti, Ana B. T. Dias, Miguel M. Burnier, Fadi Brimo, Wassim Kassouf

**Affiliations:** 1 Division of Urology, Department of Surgery, McGill University, Montreal, Quebec, Canada; 2 Department of Urology, Queen’s University, Kingston, Ontario, Canada; 3 Biomedical and Molecular Sciences, Queen’s University, Kingston, Ontario, Canada; 4 Henry C. Witelson Ocular Pathology Laboratory, McGill University, Montreal, Quebec, Canada; 5 Department of Pathology, McGill University, Montreal, Quebec, Canada; Centro Nacional de Investigaciones Oncologicas, SPAIN

## Abstract

Tumor-Infiltrating Lymphocytes (TILs) has been shown to be essential to predict disease outcome in several types of cancers. Moreover, the distribution of cytotoxic T lymphocytes (CD8+) and T cells in general (CD3+) have been used to establish an Immunoscore, as a new cancer prognosticator for survival in colon and lung cancer. In bladder cancer, immune activation has been shown to be associated with genomic subtypes of muscle invasive bladder cancer (MIBC). We sought to evaluate the prognostic impact of these immune cell types in MIBC patients treated with radical cystectomy. For this purpose, cystectomy sections (n = 67) with identifiable invasive margin were selected and stained for CD8^+^ and CD3^+^ tumour infiltrating lymphocytes (TILs); both tumor core (CT) and invasive margin (IM) were assessed. Immunoscore was calculated based on previously defined criteria and used to illustrate differences in survival. High density of CD8_IM_ TILs was associated with better disease-free (DFS) (*P* = 0.01) and overall survival (OS) (*P* = 0.02) whereas CD3_IM_ TILs were associated with better OS (*P* = 0.05). Immunoscore was associated with improved DFS (*P* = 0.02) and OS (*P* = 0.05). The expression of cytotoxic T cells (CD8+ T cells) in TCGA bladder cancer was also investigated from RNA-Seq profiles of 344 cases. T cell cytotoxicity associated genes (n = 113) were derived from MSig GSEA database. Luminal (n = 121) and basal (n = 68) samples were used to evaluate expression differences. Differential expression (P<0.05) of cytotoxic T cell genes was noted across different molecular subsets of bladder cancer within TCGA analysis. Our data suggests host immune system appears to play a valuable prognostic role in MIBC.

## Introduction

Tumor cells have the ability to interact and modulate the immune system, which leads to an imbalance between tumor growth and host surveillance. These immunomodulations are crucial for clinical management of cancer, as it is also associated with development of anti-tumor drug resistance. In fact, there is increasing literature showing that the tumor microenvironment plays an important role in disease progression with the presence of Tumor-Infiltrating-Lymphocytes (TILs) being associated with favourable prognosis in various types of cancers [1, 2]. This is particularly relevant in bladder cancer given that immunotherapy, in the form of BCG, is part of the standard of care for high-risk non-muscle invasive cancer. Moreover, recently, immune checkpoint blockade therapies such as PD-L1 inhibitors have also been shown to benefit patients with metastatic bladder cancer who have failed chemotherapy[3, 4]. The association between TIL density and outcome in bladder cancer has been studied but with inconsistent results [5–8]. One reason for these discrepancies might be the fact the distribution or spatial organization of TILs within the tumor epithelial and stromal compartments are not considered which could have an important role as prognostic indicators. Whether the immune cells are concentrated in the core of the tumor tissue or at the tumor margin seems to have stronger predictive value, and this has been shown in other types of cancer[9, 10].

It is known that patients with the same TNM stage can have different clinical outcomes following surgical resection. One major limitation of this classification method is that it fails to take into account the patient anti-tumour immune response. Given the heterogeneous outcomes after cystectomy, there is need for improvement of prognostic and predictive markers for surveillance and assignment of other therapies.

Incorporating the number, type, and distribution of immune cells, Galon et al derived a simple classification system called the “Immunoscore”[5]. Using these three factors, a score of I0 to I4 is given to the tumor specimen. A higher score represents greater T cells (CD3^+^) and cytotoxic T cells (CD8^+^) cell infiltration in the tumor in both the core and margin, and with memory T cells (CD45RO^+^) has been associated with better prognosis in colon cancer[10–12]. Therefore, we sought to evaluate the prognostic impact of lymphocyte distribution and the Immunoscore in muscle invasive bladder cancer (MIBC) patients treated with radical cystectomy.

## Materials and methods

### Patient population

Tumor blocks were retrieved from all patients who underwent radical cystectomy at the McGill University Health Center between 2011 and 2013. All consecutive cases of non-metastatic, invasive bladder cancer (pT1-T4) were included. Clinical data such as tumor stage, neoadjuvant or adjuvant chemotherapy, intravesical therapy, time to recurrence and death were collected retrospectively. Written informed consent was obtained at the time of surgery and ethics approval was obtained from the McGill University Health Center institutional review board.

### Immunohistochemistry

Formalin fixed Paraffin embedded H&E stained sections of cystectomy were initially reviewed. Slides that incorporated the invasive margin of the tumour were selected. Sections were then deparaffinised and hydrated. After antigen retrieval, the sections were incubated with CD8 (C8/144B; Dako; 1:100 dilution) and CD3 (2GV6, Ventana) antibodies. ImmunoCruz mouse ABC staining system (Santa Cruz Biotechnology) for CD8 and ultraView Universal DAB detection kit (Ventana) for CD3 were used for secondary antibody. Slides were digitally conserved using the Aperio. Three non-contiguous areas of highest lymphocyte density were selected at both the CT and IM. The number of CD3^+^ and CD8^+^ TILs in each selected area (CD3_CT_, CD3_IM_, CD8_CT_, CD8_IM_) were estimated using Aperio image analysis software. The average of three non-contiguous areas was used to eliminate sampling error[11, 13]. The reviewer who selected the areas for analysis was blinded to the clinical outcome of the patient.

### Immunoscore

The Immunoscore (I0 to I4) was calculated based on the density of CD3^+^ and CD8^+^ TILs in both the CT and IM of the tumor. For example, if both markers were elevated in both CT and IM, the highest score of I4 was given. If one marker was high in CT but was low in IM while the other marker was high in both regions, then a score of I3 was given. Similar method was applied to I2 and I1. Lastly, if both markers were low in both regions, then the lowest possible score of I0 was given. The “minimum P value” approach was used to define the cut-off point between high vs. low density for each specimen. The cut-off estimation was determined using the freely available bio-informatics tool X-Tile, for biomarker assessment that uses a minimal p-value approach for outcome-based cut-off optimization[14]. Based on previous studies, the lowest score has been linked to the worst prognosis[5]. This methodology was selected in accordance to previously established definitions used by Galon *et al*. in colon cancer[5].

### The Cancer Genome Atlas (TCGA)

We recently reported the immune classification of TCGA MIBC tumours[15]. The publicly available global transcriptomic sequencing (RNA-Seq) data from 412 MIBC cases, with the corresponding clinical information was downloaded from TCGA data portal (https://gdc-portal.nci.nih.gov/), now part of the National Cancer Institute’s Genetic Data Commons. From a cohort of 344 previously untreated MIBC cases we further selected those cases that specifically belonged to the luminal (n = 121) and basal (n = 68) subtypes. From the MSig database a list of 113 T cell cytotoxicity associated genes were selected and their expression levels evaluated between the luminal and basal subtypes.

### Statistics analysis

In the TCGA analyses, differences in gene expression were evaluated using R statistical environment (Bioconductor) as described previously[16]. Benjamin-Hochberg false discovery correction was applied. A p-value of <0.05 was considered statistically significant.

Clinical outcomes were correlated with Immunoscore. DFS and OS were analyzed using Kaplan-Meier plots. Significance among patient groups was calculated by using the log-rank test. Multivariate Cox proportional hazards model was used to determine HR. All tests were two-sided, and all analyses were done using the statistical software STATA version 13 (STATA Corp., TX, USA). P < 0.05 was considered statistically significant.

## Results

### Clinico-pathologic and socio-demographic characteristics of patient cohort

To determine the spatial organization of TILs, the distribution and density of CD3^+^ and CD8^+^ TILs were evaluated in an archival of radical cystectomy specimens. As shown in Table 1, a total of 67 patients were included in the study population with a mean follow up of 21.9 months for patients alive (median 15 months, IQR 32.5 months). The median age of the cohort is 68.9 (IQR 17.5) years. T stage was distributed as follows: pT1 (7.5%), pT2 (16.4%), pT3 (55.2%), and pT4 (20.9%). Overall, 21% of patients received neoadjuvant chemotherapy, 20% received adjuvant chemotherapy. Previous intravesical BCG was given in 26.1% of patients and did not affect density of CD3^+^ or CD8^+^ in neither the tumor core nor invasive margin (P > 0.05 in all groups).

**Table 1:** Patient characteristics.

Variable	Categories	Patients (*n* = 67)	
		No^a^	%
**Age, years at reference date**	<65	26	38.8
	> 65	41	61.2
	Median (IQR)^b^	67.5 (17.5)	
**Gender**	Male	51	75.4
	Female	16	24.6
**Stage (T)**	pT1	5	7.5
	pT2	11	16.4
	pT3	37	55.2
	pT4	14	20.9
**Stage (N)**	N0	41	61.2
	N1	12	17.9
	N2	9	13.4
	N3	5	7.5
**Margins**	No	60	89.5
	Yes	7	10.4
**LVI**	No	20	29.9
	Yes	47	70.1
**CIS**	No	20	30.3
	Yes	47	60.7
**Prior intravesical therapy**	No	50	73.8
	Yes	17	26.1
**Chemotherapy (NeoAdj)**	No	51	78.5
	Yes	14	21.5
**Chemotherapy (Adjuvant)**	No	52	80.0
	Yes	13	20.0

^a^Numbers may not add to total because of missing data

^b^Interquartile range (IQR)

### Density and location of CD3^+^ and CD8^+^ TILs in MIBC correlates with disease outcome

**Fig 1:**
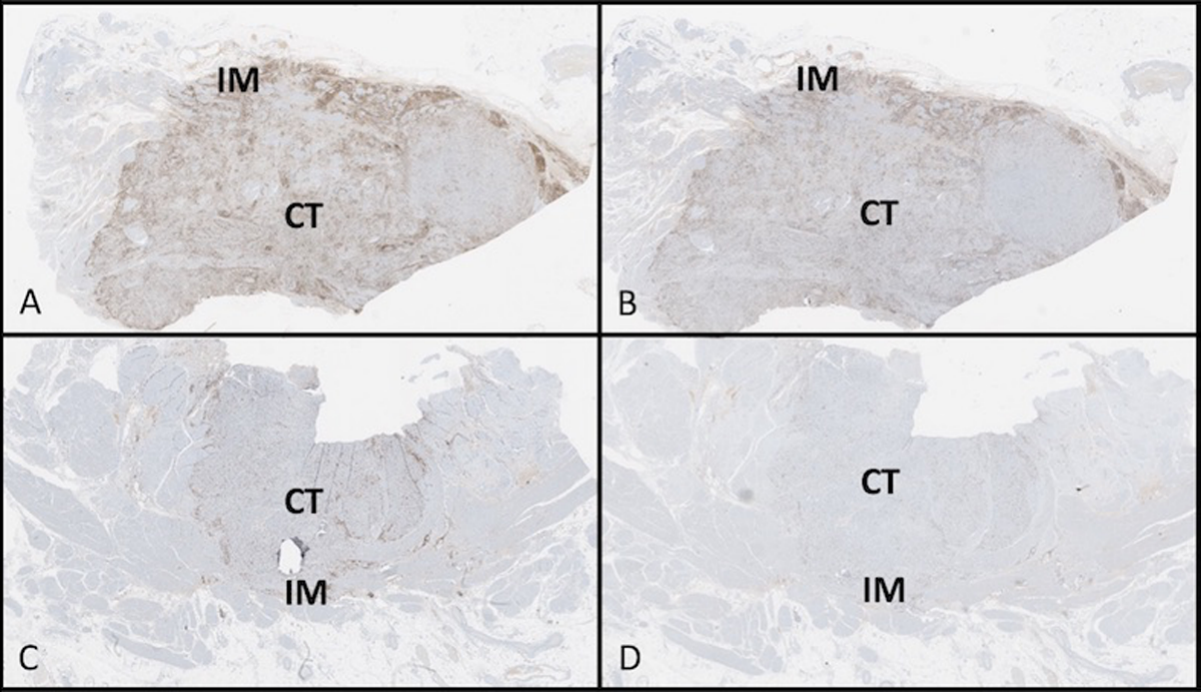
High vs. low density and locations of CD8^+^. (A) High concentration CD3^+^ in CT and IM. (B) High concentration CD8^+^ in CT and IM. (C) Low concentration CD3^+^ in CT and IM. (D) Low concentration CD8^+^ in CT and IM.

**Fig 2:**
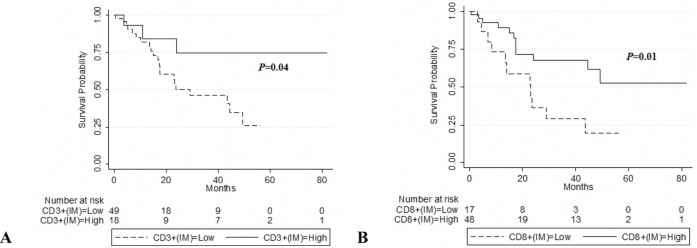
Kaplan–Meier plots of OS according to CD3+ and CD8+ TILs in the invasion margin (IM). (A) OS in all patients according to Low CD3+(IM) ≤ 290 (n = 49) vs. High CD3+(IM) > (n = 18). (B) OS in all patients according to Low CD8+(IM) ≤ 70 (n = 17) vs. High CD8+(IM) > 70 (n = 48). P values are from log-rank tests.

**Fig 3:**
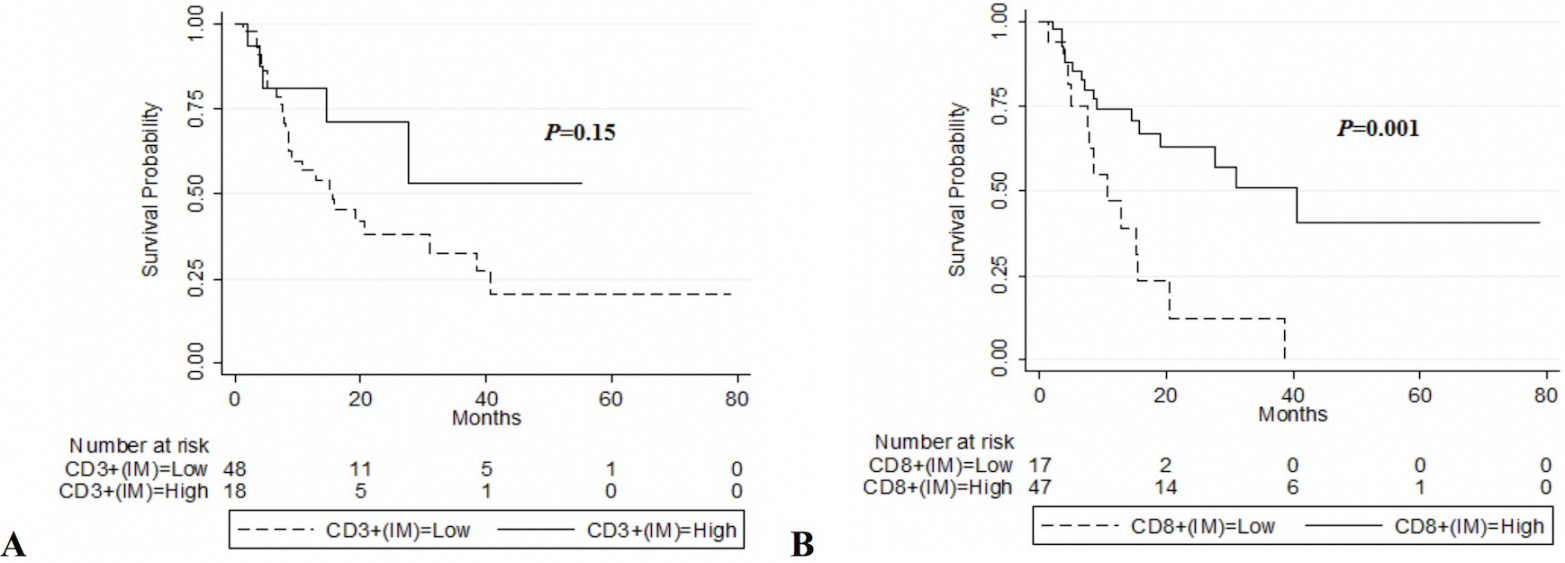
Kaplan–Meier plots of DFS according to CD3+ and CD8+ TILs in the invasion margin (IM). (A) DFS in all patients according to Low CD3+(IM) ≤ 290 (n = 49) vs. High CD3+(IM) > 290 (n = 18). (B) DFS in all patients according to Low CD8+(IM) ≤ 70 (n = 17) vs. High CD8+(IM) > 70 (n = 48). P values are from log-rank tests.

To assess the predictive potential of CD3^+^ or CD8^+^ cell densities in different tumour regions (CT and IM), the patients were divided into two groups using the minimum *P*-value cut-off values for CD3^+^ or CD8^+^ densities in each tumour region (290, 490, 70, and 116 cells per mm^2^ for CD3_IM_, CD3_CT_, CD8_IM_ and CD8_CT_, respectively). Representative sections showing high and low density of CD3^+^ and CD8^+^ TILs are displayed in Fig 1. High CD8^+^ in the IM was associated with prolonged OS (*P* = 0.01) (Fig 2B) and DFS (*P* = 0.001) (Fig 3B). Similar results were found for CD3^+^ TILs with regards to OS (*P* = 0.04) but these results did not meet statistical significance for DFS (*P* = 0.15) (Figs 2A and 3A).

After controlling for pathologic stage, surgical margin, prior BCG administration and use of peri-operative chemotherapy, higher levels of CD8^+^ TILs in the invasive margin independently associated with better outcomes (OS: HR 0.29, 95% CI 0.10–0.95, *P* = 0.04, DFS: HR 0.35, 95% CI 0.14–0.86, *P* = 0.02) (Tables 2 and 3). Higher Immunoscore independently associated with DFS (HR 0.13, 95% CI 0.02–0.76, *P* = 0.02) (Table 3).

**Table 2:** Multivariable analysis of factors predicting OS.

OVERALL SURVIVAL (OS)					
		Univariate HR (95% CI)	*P*	Multivariate (95% CI)	*P*
Organ-confined^a^	Yes	1.0 (REF)		1.0 (Ref)	
	No	4.5 (1.05–19.3)	*0*.*04*	1.36 (0.23–8.15)	*0*.*73*
Surgical margin	No	1.0 (REF)		1.0 (Ref)	
	Yes	2.54 (0.93–6.95)	*0*.*07*	10.9 (2.38–50.7)	*0*.*002*
LVI	No	1.0 (Ref)		1.0 (Ref)	
	Yes	3.27 (1.21–8.86)	*0*.*02*	6.89 (1.22–38.9)	*0*.*03*
Intravesical Therapy	No	1.0(REF)		1.0 (Ref)	
	Yes	0.96 (0.38–2.44)	*0*.*93*	2.48 (0.81–7.60)	*0*.*11*
Chemotherapy^b^	No	1.0 (Ref)		1.0 (Ref)	
	Yes	0.38 (0.17–0.90)	*0*.*03*	0.49 (0.15–1.54)	*0*.*22*
Immune marker					
CD3+CT	Low	1.0 (REF)		1.0 (REF)	
	High	0.67 (0.30–1.50)	*0*.*33*	2.91 (0.85–10.0)	*0*.*09*
CD3+IM	Low	1.0 (REF)		1.0 (REF)	
	High	0.30 (0.09–1.01)	*0*.*05*	0.57 (0.12–2.63)	*0*.*47*
CD8+CT	Low	1.0 (REF)		1.0 (REF)	
	High	0.53 (0.22–1.25)	*0*.*15*	0.32 (0.09–1.11)	*0*.*07*
CD8+IM	Low	1.0 (REF)		1.0 (REF)	
	High	0.37 (0.16-.083)	*0*.*02*	0.33 (0.12–0.88)	*0*.*03*
Immunoscore	0	1.0 (REF)		1.0 (REF)	
	1	0.57 (0.05–6.48)	*0*.*65*	0.96 (0.80–11.5)	*0*.*97*
	2	0.48 (0.10–1.27)	*0*.*35*	1.04 (0.19–5.68)	*0*.*96*
	3	0.18 (0.25–1.27)	*0*.*08*	0.15 (0.01–1.68)	*0*.*12*
	4	0.20 (0.04–0.98)	*0*.*05*	0.51 (0.09–2.90)	*0*.*45*

^a^Organ-confined: ≤T2N0; Non organ-confined: T3-4 or N+

^b^Chemotherapy: Neoadjuvant and/or adjuvant

**Table 3:** Multivariable analysis of factors predicting DFS.

Disease Free Survival (DFS)					
		Univariate HR (95% CI)	*P*	Multivariate (95% CI)	*P*
Organ-confined^a^	Yes	1.0 (REF)		1.0 (REF)	
	No	1.43 (0.54–3.78)	*0*.*47*	1.50 (0.34–6.67)	*0*.*60*
LVI	No	1.0 (REF)		1.0 (REF)	
	Yes	1.11(0.50–2.50)	*0*.*79*	0.57(0.16–2.00)	*0*.*38*
Surgical margin	No	1.0 (REF)		1.0 (REF)	
	Yes	0.95 (0.39–2.31)	*0*.*91*	1.06 (0.37–3.05)	*0*.*91*
Intravesical Therapy	No	1.0 (REF)		1.0 (REF)	
	Yes	1.19 (0.52–2.76)	*0*.*67*	1.91 (0.70–5.23)	*0*.*21*
Chemotherapy^b^	No	1.0 (REF)		1.0 (REF)	
	Yes	0.59 (0.28–1.21)	*0*.*15*	0.63 (0.26–1.55)	*0*.*32*
Immune marker					
CD3+CT	Low	1.0 (REF)		1.0 (REF)	
	High	0.73 (0.36–1.51)	*0*.*40*	1.27 (0.46–3.53)	*0*.*64*
CD3+IM	Low	1.0 (REF)		1.0 (REF)	
	High	0.50(0.19–1.31)	*0*.*16*	0.60 (0.16–2.17)	*0*.*43*
CD8+CT	Low	1.0 (REF)		1.0 (REF)	
	High	0.76 (0.33–1.72)	*0*.*51*	0.55 (0.18–1.65)	*0*.*29*
CD8+IM	Low	1.0 (REF)		1.0 (REF)	
	High	0.31 (0.15–0.68)	*0*.*01*	0.35 (0.14–0.86)	*0*.*02*
Immunoscore	0	1.0 (REF)		1.0 (REF)	
	1	0.35 (0.05–2.53)	*0*.*30*	0.42 (0.05–3.23)	*0*.*41*
	2	0.31 (0.06–1.46)	*0*.*14*	0.31 (0.07–1.52)	*0*.*15*
	3	0.21 (0.04–1.06)	*0*.*06*	0.20 (0.03–1.14)	*0*.*07*
	4	0.14 (0.03–0.69)	*0*.*02*	0.13 (0.02–0.76)	*0*.*02*

^a^Organ-confined: ≤T2N0; Non organ-confined: T3-4 or N+

^b^Chemotherapy: Neoadjuvant and/or adjuvant

### Determination of CD8 TIL infiltration in TCGA Bladder cancer transcriptome profiling dataset

Four studies of MIBC have identified transcriptional subtypes of MIBC [17–20] with considerable overlap [21]. There are two major subtypes similar to the "Basal" and "Luminal" subtypes of breast cancer with distinct therapeutic responses[22] and potentially with some predictive ability. Therefore, we sought to look at the pre-treatment gene expression of the immune tumor microenvironment in the TCGA data to explore the significance of CD8 TIL infiltration.

**Fig 4:**
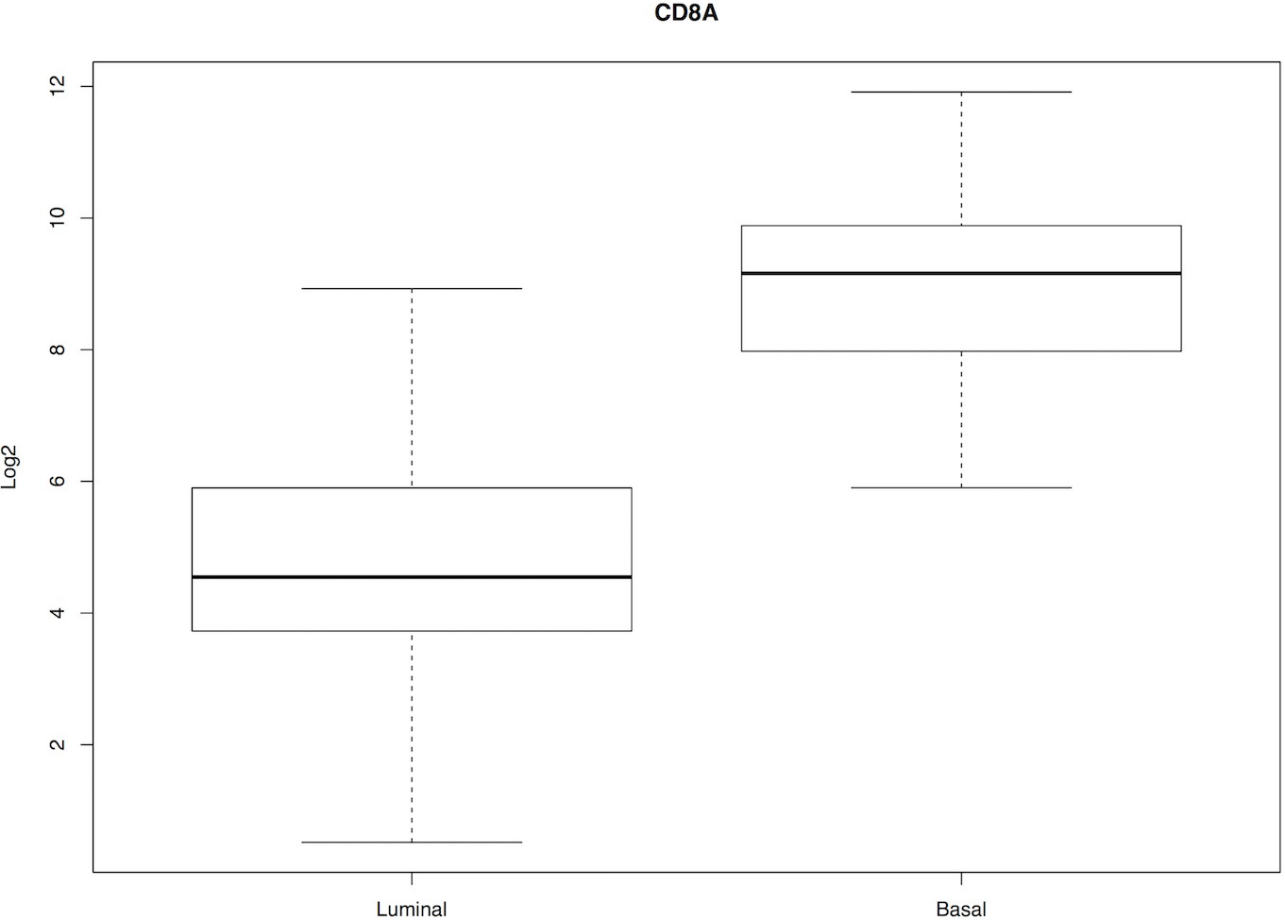
Box plots showing differential expression of *CD8A* transcripts (*p<0*.*05)* in luminal (n = 121) vs basal (n = 68) MIBC tumours.

**Fig 5:**
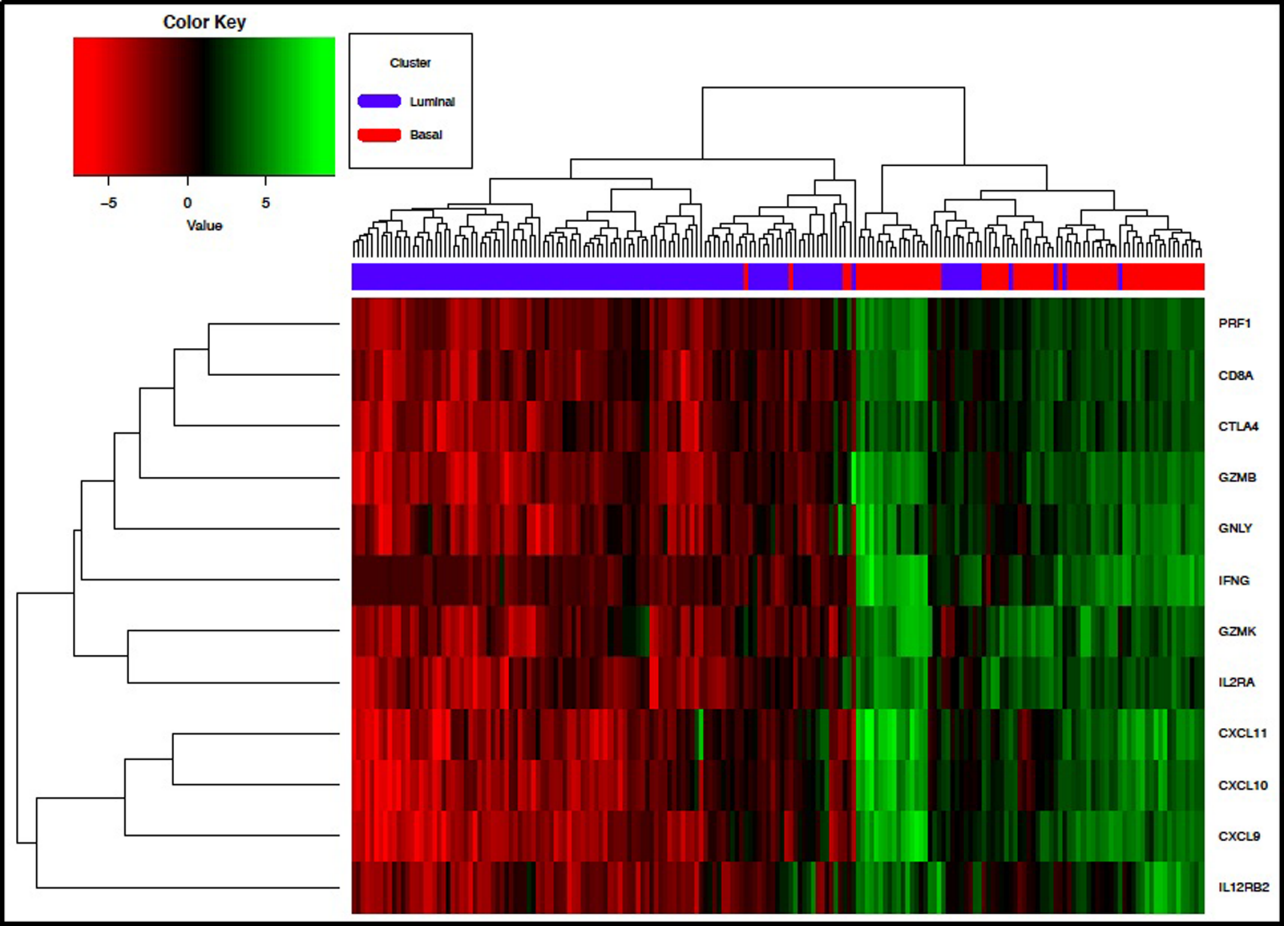
Heatmap summary showing the significantly differentially expressed T cell cytotoxicity associated genes between TCGA (Blue) clusters I (Luminal n = 121) and (Red) cluster IV (Basal n = 68). Red indicates low expression and green indicates high expression.

**Fig 6:**
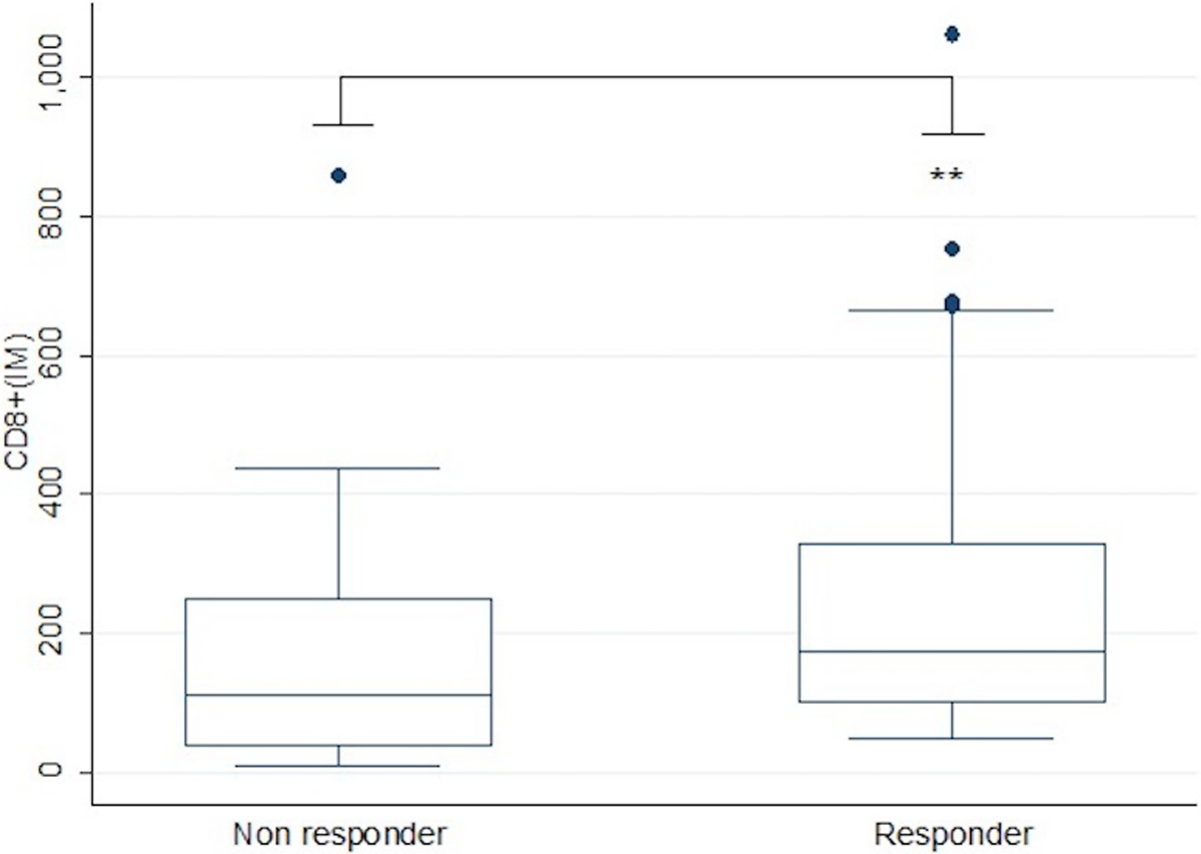
Box plots showing significantly differential CD8^+^(IM) infiltration among non responders and responders (*P* = 0.01) to neoadjuvant chemotherapy.

In the current study, we included profiles from only the clusters I and IV, which represent the two extreme pre-existing scenarios of luminal and basal subtypes, and are defined as T-cell inflamed and non-inflamed, respectively. These analyses revealed significantly higher expression of *CD8A* transcripts (*p<0*.*05)* among basal subtype in comparison with luminal MIBC (Fig 4). Moreover, higher expression of cytotoxicity T cell genes was also associated with basal subtypes (Fig 5). Interestingly, a sub analysis of CD8^+^ TILs in our patient population who received neoadjuvant chemotherapy revealed a significant association (*P* = 0.01) with chemotherapy response only when CD8^+^ TILs were infiltrated into the invasive margin, with higher density of CD8_IM_ among responders (Fig 6).

## Discussion

Previous studies looking at TILs and bladder cancer prognosis have yielded conflicting results [6–8, 23]. Higher density of CD3^+^ and CD8^+^ TILs has both been associated with improved survival[24, 25]. For instance, presence of intratumoral CD8^+^ TILs has been shown to be correlated with improved DFS after cystectomy[6] with a median DFS significantly longer in those with higher CD8^+^ TILs within the tumor (>80 months vs. 13 months P < 0.001). However, the authors did not specify which region of the tumor was examined. In contrast, this conclusion was challenged by other studies that have failed to establish TILs as a prognostic marker in bladder cancer[7, 8].

It is necessary to note that none of these studies differentiated lymphocytes taken from CT vs. IM, that may explain the inconsistent results among the studies. There is increasing evidence that the distribution of lymphocytes between the tumor core and invasive margin needs to be addressed since their influence on clinical outcome can differ. One study on colon cancer showed that patients with high CD45RO^+^ in both the CT and IM regions had better DFS and OS survival compared to those with mixed patterns; such as high concentration of lymphocytes in CT and low in IM, or low CT and high IM[12]. Likewise, another study on hepatocellular carcinoma showed that the concentration of lymphocytes in the CT is more predictive of outcome than those in IM[10].

Our study is the first to differentiate CT and IM when looking at the influence of TILs on prognosis after cystectomy. We showed that CD8_IM_ is a strong independent predictor of disease recurrence even after controlling for stage, lymphovascular invasion and use of peri-operative chemotherapy and BCG. This illustrates that lymphocyte distribution within the tumor mass is more important than just the presence of TILs. Our data showed that the density of cytotoxic lymphocytes at the IM seems to have stronger predictive value than those in the CT. It is hypothesized that the immune cells at the margin of invasion may act as a protective barrier against micrometastasis, which may explain why a higher lymphocyte density is associated with better prognosis regardless of tumor stage.

In addition to TIL distribution, we found that TIL subtype is another important predictive factor. While CD8^+^ lymphocyte correlated strongly with recurrence and survival, even after controlling for potential confounders, the prognostic value of CD3^+^ was less, which might be explained by the fact CD3^+^ includes CD4^+^ TILs, that does not have significant prognostic value. Kaplan-Meier curves show that CD3_IM_ was associated with better prognosis but this effect is lost on multivariate analysis. However, larger sample size is needed to validate the prognostic impact of CD3^+^. Also, previous studies on colon cancer have used effector memory T cell CD45RO^+^ in combination with CD8^+^ to calculate the Immunoscore[12, 26, 27]. CD45RO^+^ is a marker worth exploring in future studies, and may be more specific for bladder as other CD8^+^ TIL activation marker such as IFNg as well.

The Immunoscore takes into account the distribution of TILs between CT and IM and has been shown to have strong predictive value in other types of cancers, such as colon[11]. In a cohort of 602 colon cancer patients, those with a high Immunoscore (I4) had 4.8% relapse rate at 5 years, compared to 86% relapse in those with a low Immunoscore (I0)[12]. There was also a significant difference in survival (72% vs. 27.5% respectively)[12]. There is support towards integrating immune markers into the traditional TNM staging classification[5, 26, 28]. Validation of the Immunoscore as a new approach for the classification of colon cancer is ongoing[5]. Our results are consistent with those previously shown in colon cancer; that a higher immunoscore (I4) is associated with better OS and DFS. I4 remained an independent predictor for DFS even after adjusting for clinico-pathological risk factors.

Recently, the tumor-infiltrating immune cells have been evaluated, *in silico*, over the RNA seq data across several types of cancer from TCGA[29]. Particularly in bladder cancer, CD8^+^ T-cell infiltration had a significantly different distribution in comparison with normal adjacent bladder tissue. However, CD8^+^ T-cells infiltration was not associated with prolonged survival. On the other hand, in our analysis, the TCGA data suggests there is strong delineation of *CD8A* transcript expression between basal and luminal tumors, which could be interpreted as opposite to our findings since MIBC basal tumors are known to have worse outcomes. As per our previous report the IFNg potentially from T cell activation or other sources induces PD-L1 and IDO1 and potentially contribute to aggressive disease and worse outcomes in basal subtype tumours[16]. In contrast, distinct basal and luminal subtypes of MIBC have been identified and characterized with different sensitivity to frontline chemotherapy[17, 18]; basal being more chemosensitive than luminal. In this sense, it might be consistent with our results, considering more than 40% of our cohort patients received perioperative chemotherapy. Indeed, this was supported by the association of CD8_IM_ among responders within our cohort. As reported in ovarian cancer[30], it is possible that the higher pre-existing CD8^+^ TILs in the TME are activated and mediate their anti-tumour effect as a result of immunogenic cell death induced by the chemotherapy. These findings reinforce the importance the Immunoscore, as it adds the relevant spatial distribution component to the unidirectional immune "infiltrate" status observed from the TCGA data.

While this is the first study to evaluate the prognostic value of Immunoscore on bladder cancer, there are some limitations to highlight. Although a multivariable analysis was performed to adjust for known confounding variables, bias may remain due to the retrospective nature of the study and its limited sample size. These findings warrant validation in a larger multicenter study. Furthermore, whether the Immunoscore can be predictive for response to immunotherapy (such as PDL-1 inhibitors) in bladder cancer remains unknown.

## Conclusions

In summary, our study showed that the Immunoscore is a potential predictor of clinical outcomes after cystectomy. A strong immune response at the tumor margin is independently associated with better DFS and OS. More studies are needed to further assess the role of immune markers in bladder cancer prognosis and to determine the value of incorporating these markers in the traditional TNM staging.
